# Agricultural Detection of Norovirus and Hepatitis A Using Fecal Indicators: A Systematic Review

**DOI:** 10.1155/2021/6631920

**Published:** 2021-01-04

**Authors:** Courtney P. Victor, Karen Ellis, Frederica Lamar, Juan S. Leon

**Affiliations:** Hubert Department of Global Health, Rollins School of Public Health, Emory University, 1518 Clifton Road NE, Atlanta, GA 30322, USA

## Abstract

Fresh-produce consumers may be at risk of pathogen infection due to fecal contamination of the agricultural environment. Indicators of fecal contamination may be used as a proxy to evaluate the potential presence of human pathogens, such as norovirus and hepatitis A, on agricultural samples. The objective of this systematic review was to determine whether the presence of human norovirus or hepatitis A was associated with microbial indicators in agricultural samples including fresh produce, equipment surfaces, and hands. Four databases (Embase, PubMed, Web of Science, and Agricola) were systematically searched and fifteen articles met inclusion and exclusion criteria. After data extraction, individual indicator-pathogen relationships were assessed using Cohen's Kappa coefficient. The level of agreement between norovirus with adenovirus was 0.09 (*n* = 16, 95% CI −0.05, 0.23), indicating poor agreement using Landis and Koch's criterion. Similarly, the Kappa coefficient between norovirus with *E. coli* (*κ* = 0.04, *n* = 14, 95% CI −0.05, 0.49) or total coliforms (*κ* = 0.03, *n* = 4, 95% CI −0.01, 0.02) was also poor. The level of agreement between hepatitis A with adenovirus (*κ* = −0.03, *n* = 3, 95% CI −0.06, 0.01) or fecal coliforms (*κ* = 0, *n* = 1, 95% CI 0, 0) was also poor. There were moderate relationships between hepatitis A with *E. coli* (*κ* = 0.49, *n* = 3, 95% CI 0.28, 0.70) and total coliforms (*κ* = 0.47, *n* = 2, 95% CI 0.47, 0.47). Based on these limited results, common indicator organisms are not strong predictors of the presence of norovirus and hepatitis A virus in the agricultural environment.

## 1. Introduction

Globally, the number of produce-associated outbreaks has been increasing due to increased consumption of fresh produce [1]. For example, between 1998 and 2013, there were a total of 972 outbreaks of food-borne illness associated with fresh produce in the United States, alone [2]. Two major etiological contributors to produce-associated outbreaks are human norovirus and hepatitis A virus. According to a systematic review of viral outbreaks associated with fresh produce globally, norovirus and hepatitis A virus caused 48.7% and 46.1% of all outbreaks [3]. Norovirus is the leading etiologic agent for food-borne outbreaks associated with produce in the US and the European Union [1]. Specifically, from 1998 to 2013, it was the etiologic agent for over half of the 972 US outbreaks associated with fresh produce [2]. There were a total of 96 outbreaks of hepatitis A between 1998 and 2017, and fresh fruits and vegetables were the most common sources [4]. As these outbreaks have a significant public health and economic burden, this highlights the need for better microbiological safety practices, including detection of these pathogens in an agricultural setting or in food handlers to prevent outbreaks from occurring downstream in the produce value chain [5, 6].

Two critical steps to identify norovirus and hepatitis A in the agricultural setting are elution of the virus from the matrix and viral detection. Elution of these viruses is often difficult because food samples often have a low concentration of viruses [7] and thus complex filtration equipment, elution, and concentration methods are required to process large samples [8]. Viral detection is also difficult and falls under two categories: immunological and molecular [9]. Immunological methods, such as enzyme immunoassays (ELISA), are not commercially available for use outside of clinical settings [7]. The most validated molecular method is the real-time polymerase chain reaction (PCR), and it has been accredited by the International Standards Organization as the standard detection method for hepatitis A virus and norovirus [10]. PCR presents a challenge for pathogen detection in agricultural settings, as it requires high technical expertise, substantial time, and funds to complete. Thus, PCR is not a feasible detection method for norovirus and hepatitis A by small-scale farmers and other food producers with little background in microbiology.

Due to the low levels of norovirus and hepatitis A in the environment, indicator organisms have often been used as a proxy for their contamination [11]. A systematic review conducted in 2018 that tested fresh produce for pathogen contamination found the median result of detection to be 0% [12]. Common indicator organisms that have been suggested and tested as proxies for norovirus or hepatitis A include *E. coli*, total coliforms, fecal coliforms, enterococci, coliphage, and adenovirus. Advantages of testing for these organisms instead of norovirus or hepatitis A include faster time-to-result and lower cost. However, there are many caveats when using indicator organisms for measures of safety:

- Although the presence of fecal indicator organisms may suggest the presence of pathogens, it is important to acknowledge that this is only an inference and not a direct measure.
- While the use of indicator organisms has been mandated by regulatory agencies such as the U.S. Public Health Service and WHO for over 100 years, the methods are still widely variable and many have not been developed to specifically monitor environmental samples such as food and water [11]. Thus, it can be difficult to select appropriate indicator organisms that can approximate the risk of norovirus and hepatitis A contamination in these specific settings.
- It has been demonstrated that the presence of one organism does not indicate the presence of the other organisms due to the fact that indicators, compared to norovirus and hepatitis A, often have shorter persistence in the environment and are more susceptible to heat and pH [13].
- Bacterial indicators can multiply on foods which are in contrast to pathogens such as norovirus and hepatitis A which cannot replicate without a living host [7].
- Additionally, in a review of the correlation between indicators and their pathogens over a 40-year time period, Wu and colleagues concluded that the use of common indicators (e.g., coliphages, *E. coli*, fecal coliforms, and total coliforms) is not reliable for estimating pathogen prevalence (e.g., norovirus and hepatitis A) in water samples [14].

In summary, the utility of these organisms for inferring the presence of norovirus or hepatitis A in agricultural samples (e.g., irrigation water) is not well described. Thus, there is a need to assess whether utilizing indicator organisms is sufficient for describing the presence of norovirus and hepatitis A virus in an agricultural setting. The goal of this systematic review was to determine whether the presence of human norovirus and hepatitis A virus can be associated with common microbial indicator organisms in environmental samples (e.g., produce, water, soil, equipment, and hands).

## 2. Materials and Methods

### 2.1. Search Strategy

Electronic searches were performed using Embase, PubMed, Agricola, and Web of Science between 1994 and April 2018. The literature search was conducted independently by two investigators using the search strings displayed in Table 1.

### 2.2. Selection Criteria

**Figure 1:**
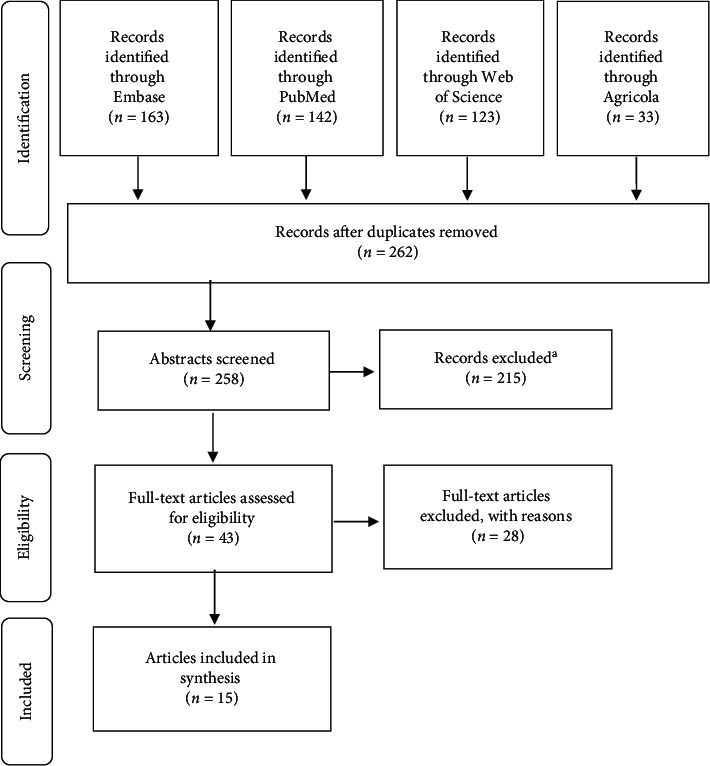
PRISMA systematic review methods. ^a^Reason described in Methods.

Following the completion of electronic searches and the removal of duplicates, 262 articles remained for screening by applying inclusion and exclusion criteria defined by the investigators on the article abstract. During the screening of the article abstract, articles were included if they were written in English, published in 1994 or later (after the development of molecular testing), addressed either human norovirus or hepatitis A virus confirmed by PCR or culture techniques, and discussed the use of indicators specifically with one of the two viruses of interest. All studies detailing laboratory experiments were excluded. To confirm the eligibility of the article, the entire full-text was reviewed to confirm that the articles must have examined the presence of indicators in water, produce, soil, equipment, or hands of workers. Since the goal of this research was to assess the relationship between norovirus and hepatitis A in agricultural settings, water was defined as any source of water that could be used for irrigation purposes. Thus, water sample types included farm pond, wells, surface water, creeks, rivers, springs, municipal clean water (effluent and sludge), and wastewater runoff. Saltwater and municipal wastewater influent were excluded as these are not water sources typically used for irrigation. Studies evaluating the removal of pathogens via treatment with antimicrobials were also included. There was no exclusion based on geography. This process is displayed in Figure 1.

### 2.3. Data Extraction

Data was extracted independently by two reviewers (K. E., F. L.) and reviewed by a third reviewer for errors (C. V.) from the selected articles. Extracted data included article title, author(s), date published, country of study, setting (urban or rural), sample setting, sample location, produce type, pathogen(s) tested, indicator(s) tested, pathogen testing method, indicator testing method, sample size, and results from pathogen-indicator tests (e.g., prevalence). In an article where more than one pathogen was assessed (i.e., norovirus and hepatitis A), results were recorded as separate observations. For some articles, authors were contacted for clarification of sample size and results, and some authors generously shared their data.

### 2.4. Data Analysis

Statistical analyses were performed using OpenEpi (http://www.OpenEpi.com), an open-source data analysis software. Interrater agreement between the presence of indicators and the presence of pathogens was evaluated using Cohen's Kappa coefficient (*κ*), where *ρ*_0_ indicates the observed agreement and *ρ*_*e*_ indicates the probability of chance agreement.

(1) $$\begin{array}{c} \kappa \equiv \frac{\rho_{0}-\rho_{e}}{1-\rho_{e}}=1-\frac{1-\rho_{0}}{1-\rho_{e}}. \end{array}$$

Kappa statistics were calculated for each pathogen and indicator described in each of the articles. Measures from the same indicator and pathogen relationship were then combined using a weighted average. Although Kappa coefficients are a direct measure, 95% confidence intervals were calculated to show the range of the estimates rather than imply statistical significance. The average Kappa coefficients were interpreted as follows: <0 indicates no agreement, 0.01–0.20 indicates slight or poor agreement, 0.21–0.40 indicates fair agreement, 0.41–0.60 indicates moderate agreement, 0.61–0.80 indicates substantial agreement, and 0.81–1.00 indicates almost perfect agreement [15]. Further, a negative Kappa indicates that the agreement is worse than what is expected by chance [15].

## 3. Results and Discussion

### 3.1. Results

The initial search resulted in a total of 461 articles from the four databases (Embase [163 articles], PubMed [142 articles], Web of Science [123 articles], and Agricola [33 articles]). There were 262 articles after duplicates were removed. After the screening of abstracts, 215 articles were removed per the inclusion and exclusion criteria outlined in the methods section. Following a screening of full-text articles, a total of 15 articles remained to be included (Figure 1).

The 15 articles that were included represented a wide geographical range and incorporated various sample types that are representative of potential sources of contamination in agricultural settings (Table 2). Three articles were sampled from the United States and Canada, five articles were sampled from Europe, four articles were sampled from Asia, one article was sampled from Africa, and two articles were sampled from Latin America. Sampling locations included farms, food processing plants, and markets. Wastewater and drinking water treatment plants and surface waters were also included to account for the potential use of these sources as irrigation water (Materials and Methods). Many included sample types were liquids, which included produce and hand rinses from farmworkers. 14 articles tested for norovirus, genogroup I, II, or both, and six articles tested for hepatitis A. All samples were aggregated according to the pathogen tested in order to calculate a median prevalence of norovirus and hepatitis A across all included articles. Across all sample types, the weighted median prevalence of norovirus was 11.82% (*n* = 1877, IQR 1.65–19.07%) and the weighted median prevalence of hepatitis A virus was 0% (*n* = 687, IQR 0–3.02%). The most commonly tested indicator organisms were human adenovirus (*n* = 16) and *E. coli* (*n* = 11). Notably, total coliforms were only tested in two articles and fecal coliforms were only tested in three articles.

To determine if there were significant relationships between these pathogens and any indicator, we measured the interrater reliability using Cohen's Kappa coefficient to determine how related the viruses were with each of the potential indicator organisms (Table 3). Two articles [21, 22] specifically assessed the relationship between norovirus and the presence of fecal indicators but did not include data in the format needed to calculate Cohen's Kappa coefficient. León-Félix and colleagues found that there was no statistical relationship between the presence of norovirus and fecal coliforms or *E. coli* [21]. López-Gálvez and colleagues did find a correlation between norovirus GI and *E. coli* (r = 0.68), but no relationship between norovirus GII and *E. coli* [22]. Using Cohen's Kappa coefficient, there were no pathogen-indicator pairs that indicated better moderate agreement (0.41–0.60) according to the evaluation criteria used (Materials and Methods). The highest correlated indicator and pathogen was the relationship between hepatitis A and *E. coli* (*κ* = 0.49, 95% CI 0.28, 0.70), followed by the relationship between hepatitis A and total coliforms (*κ* = 0.47, 95% CI 0.47, 0.47). Notably, the total number of samples tested for hepatitis A and *E. coli* was 24, and the total number of samples tested for hepatitis A and total coliforms was 15. The highest correlated indicator for norovirus was polyomavirus (*κ* = 0.21), which is a human pathogen that causes progressive multifocal leukoencephalopathy (PML) and thus not a useful environmental indicator organism [30]. The level of agreement between norovirus with adenovirus was 0.09 (*n* = 16, 95% CI −0.05, 0.23) which indicates poor agreement. The agreement between norovirus and total coliforms was also poor (*κ* = 0.01, *n* = 2, 95% CI −0.01, 0.02), as was the level of agreement between norovirus and E. coli (*κ* = 0.04, *n* = 14, 95% CI −0.05, 0.49). There was a negative interrater agreement between hepatitis A virus and adenoviruses (*κ* = −0.03, *n* = 3, 95% CI −0.06, 0.01), indicating that agreement was less than what would have been by chance. Overall, we found no significant and no better than moderate relationships between norovirus or hepatitis A and any indicator that was measured in these studies.

### 3.2. Discussion

The goal of this systematic review was to examine whether the presence of human norovirus and hepatitis A in agricultural samples was associated with indicator organisms. First, we found that the overall median prevalence across all sample types in these studies was 11.82% (*n* = 1877, IQR 1.65–19.07%) for norovirus and 0% (*n* = 687, IQR 0–3.02%) for hepatitis A. Second, there were no indicator organisms that were reliably associated with the presence of norovirus or hepatitis A in the agricultural samples evaluated in the 15 included articles.

The overall median prevalence of norovirus and hepatitis A in the agricultural environment, on our analyzed sample types, was within the range of that reported from prevalence studies. For example, Stals et al. reported the detection of 24% of norovirus RNA among produce samples (*n* = 75) from farms across Europe [31]. Shin et al. reported under 1% of norovirus RNA in various Korean agricultural samples (*n* = 773) Shin et al. [32] while, similarly, Macori et al. reported no norovirus RNA in Italian berry samples (*n* = 75). When examining hepatitis A, prevalence studies of berries [29, 32, 33], fresh vegetables and herbs [32, 34–36], and surface and irrigation water [22] also found nonexistent or low hepatitis A prevalence.

Our finding that indicator organisms are not reliably associated with pathogens in agricultural samples in our review is consistent with other findings, particularly in water research [26, 37, 38]. For example, two studies found no significant correlation between any bacterial indicators and either norovirus or hepatitis A in water samples from wastewater treatment facilities [26, 37]. A review of indicator-pathogen relationships in recreational water samples from 73 papers over a 40-year span found little evidence of the relationship between fecal indicator bacteria and viral pathogens, including norovirus and hepatitis A [38]. Additionally, our findings are also supported by individual analyses from the included articles. Tian and colleagues found no correlation between *E. coli* and norovirus in irrigation water [6]. There was also no correlation found between norovirus and indicator bacteria (*E. coli*, total coliforms, fecal coliforms, and enterococci) from groundwater, fresh produce, or hand rinses from packinghouse workers [18, 21]. Carducci and colleagues found no significant correlation between either hepatitis A or norovirus and somatic coliphages, *E. coli*, or enterococci in wastewater samples [26]. A few isolated studies did find a positive relationship between indicators and pathogen prevalence. For example, López-Gálvez and colleagues found a positive correlation between *E. coli* and norovirus GI in irrigation water [22]. In a review conducted by Wu and colleagues, it was found that the presence of coliphages, fecal streptococci, and total coliforms in water samples resulted in greater odds of enterovirus presence [14].

Many reasons can explain why we found a lack of relationship between indicator organisms and norovirus or hepatitis A. The most salient explanation is the biological differences between indicator organisms, which are common bacteria, and norovirus and hepatitis A, which are viral pathogens. These biological differences may lead to a discordance in the detection of bacterial indicators versus norovirus and hepatitis A. For example, an initial inoculum of bacterial indicators in an environmental sample may be amplified, unlike viruses, because bacteria, and not viruses, can reproduce outside the host [39]. In addition, there may be differences in environmental detection of organisms based on the timing of the initial contamination event. Specifically, the biological structure of norovirus and hepatitis A, unlike common indicators, promotes long environmental persistence (weeks to months) [40]. Further, some indicator organisms, such as total coliforms and enterococci, are not strict markers of human fecal contamination because they naturally occur in the environment in contrast to norovirus and hepatitis A which originate exclusively from human waste [40]. A second explanation may be the low sample sizes of some of the indicator-pathogen pairs and low pathogen prevalence in the agricultural environment, as has been reported in other studies [12]. In support of these points, Wu and colleagues also found in their review that sample size and pathogen prevalence were important factors in the correlation between microbial indicators and pathogens [14]. This is important, as viruses are often difficult to detect in agricultural samples, including water samples [41]. As described for recreational water, low pathogen prevalence can lead to an underestimation of health risk when using indicator organisms as proxies for pathogen presence [13].

There were several strengths in this review, related to both the methodology and the data. The search for articles and data extraction were conducted systematically by two independent reviewers (K. E. and F. L.), which reduced the potential for evidence selection bias. A third reviewer (C. V.) resolved disagreements and assured consistency in data extraction and quality. Second, the articles in the systematic review represented diversity in both geography and setting. We were able to assimilate data from Europe, Asia, and North and South America from different study sites such as lagooning inlets, rivers, and packing plants. These sites are representative of many potential sources of contamination of agricultural samples. Lastly, we utilized raw data for the analysis, in several instances requesting the raw data from the original authors, rather than summarizing statistics reported in the included manuscripts which were calculated using different methodologies.

There were also some limitations associated with this review. Due to the general acknowledgment that indicator organisms cannot reliably predict the presence of pathogens, our review could be subject to publication bias. However, we may have reduced this bias by including articles with different research objectives than ours. The goal of most of the included manuscripts was to assess the microbiological quality of agricultural samples, not to estimate the association between viruses and indicators. This is evident in the types of indicators that were included in this review (i.e., JC polyomavirus) that are not typically thought of as indicator organisms but may be more considered as index organisms. While we had a large variety of sample types included in our analysis, there were very few of the same sample type. Thus, instead of grouping the analyses by each sample type, we calculated average Kappa coefficients with data aggregated by sample type. Lastly, there were various methods used for the detection of indicator organisms, which could have led to differences in the ability to detect these organisms in agricultural samples.

## 4. Conclusions

The low interrater agreement between even the most commonly used indicator organisms and norovirus and hepatitis A suggests that while these organisms may be appropriate as indicators of microbial quality (i.e., mold, poor taste, and foul smell) [11], they cannot be reliably used as indicators of safety (situations in which the presence of an organism indicates the presence of a pathogen) [11] in an agricultural setting. To measure safety in the agricultural setting, there is a need for better viral detection methods that can be successfully employed in agricultural settings. Meanwhile, focusing on microbial quality and the prevention of fecal contamination upstream in agricultural processing as a way to mitigate risk may be the most effective strategy available to prevent outbreaks of norovirus and hepatitis A associated with produce. Since these are human-sourced pathogens, preventing feces from entering the environment, either through improved waste-management or hand hygiene, is an effective way to mitigate risk until better detection methods are developed.

## Figures and Tables

**Table 1:** Search string operations.

Database	Search string	Article yield
Embase	(Norovirus NOT murine NOT calicivirus NOT feline) OR (hepatitis NEXT/2 a NOT hepatitis NEXT/2b NOT hepatitis NEXT/2 c) AND indicator*∗* AND (water OR soil OR hand*∗* OR produce*∗* OR fruit*∗* OR vegetable*∗* OR equipment) NOT (dairy OR fish OR shellfish OR marine OR ocean*∗*)	163
PubMed	((Norovirus NOT Murine NOT calicivirus NOT feline) OR “Hepatitis A″) AND indicator*∗* AND (water OR soil OR Hand*∗* OR produce*∗* OR fruit*∗* OR vegetable*∗* OR equipment) NOT (dairy OR fish OR shellfish OR marine OR ocean*∗*)	142
Agricola	((Norovirus NOT Murine NOT calicivirus NOT feline) OR “Hepatitis A″) AND indicator*∗* AND (water OR soil OR Hand*∗* OR produce*∗* OR fruit*∗* OR vegetable*∗* OR equipment) NOT (dairy OR fish OR marine OR ocean*∗* or shellfish)	33
Web of Science	((Norovirus NOT Murine NOT calicivirus NOT feline) OR “Hepatitis A″) AND indicator*∗* AND (water OR soil OR Hand*∗* OR produce*∗* OR fruit*∗* OR vegetable*∗* OR equipment) NOT (dairy OR fish OR marine OR ocean*∗* or shellfish)	123

**Table 2:** Characteristics of included articles.

Article	Virus tested	Organisms including Indicator(s) tested	Virus testing method	Sample type	n	Study site	Country
Tian et al., 2017 [6]	Norovirus (general)	O157 and non-O157 STEC, *L. monocytogenes*, *Salmonella*, *E. coli*	qRT-PCR	Surface Water	860	Agricultural Region	United States (California coast)
D'Ugo et al., 2016 [16]	Norovirus GII & Hepatitis A	Human enterovirus, Hepatitis E, Adenovirus 41, Mammalian *Orthoreovirus*	c-DNA construction and q-PCR	River, lake, sea, dam water	15	Open spaces, grazing land, artificial areas	Italy, France, Ireland, Bulgaria, Germany, Turkey
Brassard, Gagne, Genereux, & Cote, 2012 [17]	Norovirus GI	Swine Hepatitis E virus,	RT-PCR	Produce Rinse	61	Farm	Canada
Cheong et al., 2009 [18]	Norovirus (general)	Human adenovirus, enteroviruses	Nested RT-PCR, Cell-culture based PCR	Irrigation Water, Produce Rinse	29, 30^a^	Farm	South Korea
Fernandez-Cassi et al., 2016 [19]	Norovirus GI & GII	Human adenovirus, JC polyomavirus, Heterotrophic bacteria, *E. coli*, intestinal enterococci, Arcobacter spp.	Nested RT-PCR	Lagooning inlet & outlet water	12^b^	Wastewater Treatment Plant	Spain
Phanuwan et al., 2006 [20]	Norovirus GI & GII, Hepatitis A	Total coliforms, *E. coli*, Enterovirus, Human adenovirus	TaqMan PCR	Floodwater, river water, tap water	21	Residential area	Indonesia
León-Félix, Martínez-Bustillos, Báez-Sañudo, Peraza-Garay, & Chaidez, 2010 [21]	Norovirus (general)	Fecal coliforms, *E. coli*,	RT-PCR	Hand rinse	97	Agricultural Packing House	Mexico
López-Gálvez et al., 2016 [22]	Norovirus GI & GII, Hepatitis A	*E. coli*	qRT-PCR	Irrigation water	108	Commercial Greenhouse	Spain
Montazeri et al., 201 [23])	Norovirus GI & GII	Enterovirus, Fecal coliforms, *E. coli*, Enterococci,	qRT-PCR	Effluent wastewater	24	Wastewater Treatment Plant	United States
Silverman, Akrong, Amoah, Drechsel, & Nelson, 2013 [24]	Norovirus GII	Human-specific Bacteroidales, Human adenovirus, *E. coli*,	qRT-PCR	Irrigation Water	20	Farm	Ghana
Hernandez, Monge, Jimenez, & Taylor, 1997 [25]	Hepatitis A	Fecal coliforms,	PCR	Produce rinse	10	Market	Costa Rica
Carducci et al., 2009 [26]	Norovirus GI & GII, Hepatitis A	Human adenovirus, Enterovirus, Somatic coliphages, *E. coli*, Enterococci	PCR	Effluent wastewater	29	Wastewater Treatment Plant	Italy
Haramoto et al., 2012 [27]	Norovirus (general)	Total coliforms, *E. coli*, F-specific coliphage	PCR	Effluent drinking water	184^c^	Water treatment plant	Japan
Shrestha, Shindo, Sherchand, & Haramoto, 2018 [28]	Norovirus (general)	JC and BK Polyomavirus, Pepper Mottle virus, Tobacco Mosaic virus	qRT-PCR	Irrigation water	49	River, Pond, Canal, Groundwater	Nepal
Maunula et al., 2013 [29]	Norovirus GI & GII, Hepatitis A	Human Adenovirus, Hepatitis E virus, Porcine adenovirus, Bovine polyomavirus	RT-PCR	Irrigation Water, Hands, Toilets, Door handles, Produce Rinse	36, 18, 14^d^	Berry producing farms, processing plants, and market	Finland, Poland, Serbia, Czech Republic

^a^29 samples tested from irrigation water and 30 produce rinse samples were collected. ^b^12 samples taken from each sample site. ^c^Dr. Haramoto provided raw data that included more samples that were tested than were listed in the article cited here. ^d^Dr. Maunula provided raw data for this paper that included 36 samples tested for human adenovirus, 14 samples tested for hepatitis E virus, and 18 samples tested for porcine and bovine adenoviruses.

**Table 3:** Types of pathogen/indicator analyzed by study^a^.

Pathogen	Indicator (n)^b^	Kappa coefficient^c^ (95% CI)
Norovirus^d^	Adenovirus (16)	0.09 (−0.05, 0.23)
	*E. coli* (14)	0.04 (−0.05, 0.49)
	F. coliphage (2)	0.07 (−0.07, 0.21)
	Legionella (4)	0.19 (−0.24, 0.52)
	Total coliforms (4)	0.03 (−0.01, 0.02)
	JC Polyomavirus (6)	0.39 (0.04, 0.74)

Hepatitis A	Adenovirus (3)	−0.03 (−0.06, 0.01)
	*E. coli* (3)	0.49 (0.28, 0.70)
	Fecal coliforms (1)	0 (0,0)
	Total coliforms (2)	0.47 (0.47, 0.47)

^a^Excludes León-Félix et. al and López-Gálvez et. al studies. ^b^Number of analyses that included both the pathogen and indicator of interest. ^c^Kappa coefficients were calculated and then weighted by sample size for the summary estimate. ^d^All genotypes of norovirus were grouped for this analysis.

## Data Availability

The extracted data used to support the findings in this systematic review can be found in the previously reported studies that are cited within the manuscript and summarized in Table 2. The processed data are available upon request from the corresponding author.
